# A systematic review of clinical trials on Ginkgo (*Ginkgo biloba*) effectiveness on sexual function and its safety 

**DOI:** 10.22038/AJP.2021.17813

**Published:** 2021

**Authors:** Zahra Niazi Mashhadi, Morvarid Irani, Mahin Kiyani Mask, Clara Methie

**Affiliations:** 1 *Student Research Committee, Torbat Heydariyeh University of Medical Sciences, Torbat Heydariyeh, Iran*; 2 *Department of Midwifery, School of Nursing and Midwifery, Torbat Heydariyeh University of Medical Sciences, Torbat Heydariyeh, Iran*; 3 *Health Sciences Research Center, Torbat Heydariyeh University of Medical Sciences, Torbat Heydariyeh, Iran*; 4 *Clinical Instructor Mpilo School of Midwifery Zimbabwe. Zimbabwe Confederation of Midwives, Bulawayo, Zimbabwe*

**Keywords:** Sexual dysfunction, Complementary and alternative medicine, Ginkgo (Ginkgo biloba) Systematic review

## Abstract

**Objective:**

During recent years, evidence-based practice as a way to support higher standards of care was emphasized by health care policymakers. Sexual dysfunction (SD) is a common problem that affects the quality of life in individuals. Today, the use of *Ginkgo biloba* extract (GBE) for treating SD has been considered, so this study was performed to evaluate the current evidence for the efficacy and safety of ginkgo in treating SD.

**Materials and Methods:**

In this review study, electronic databases of PubMed, Scopus, Cochrane, Google Scholar, Web of science and Persian databases such as SID and Magiran were searched up to March 2020, to identify all the studies reporting the effect of GBE for effectiveness on sexual function and its safety. The search was performed using the keywords of Ginkgo, *Ginkgo biloba*, Complementary and alternative medicine, women sexual dysfunction, and male sexual dysfunction. The quality of included studies was assessed using the Oxford Center for Evidence Based Medicine checklist.

**Results:**

Among 156 articles found in the initial search, 5 randomized controlled trials (475 participants) were selected for this study. After a meticulous review, we found that *G. biloba* can have positive effects on the sexual function of postmenopausal women, while evidence shows that it has no effect on the sexual function of antidepressants users. Headaches and gastrointestinal disturbance were among the adverse events mentioned in several trials.

**Conclusion:**

We concluded that *G. biloba* has limited positive effects on sexual function and more studies are needed to confirm these findings.

## Introduction

Sexual dysfunction (SD) is a condition that a person cannot be satisfied with his or her sexual activity during a sexual cycle (Chen et al., 2013 ▶). While it is not possible to accurately compare the rate of this disorder in men and women, evidence suggests that it is more common in women (Sharma and Kalra, 2016 ▶). The prevalence of SD in previous studies varied from 14.1 to 90.1% (Omani-Samani et al., 2019 ▶; Santana et al., 2019 ▶). Satisfaction with sexual function plays an important role in women's quality of life, so any impairment of this function can reduce their sense of well-being and quality of life (Amiri et al., 2014). Diseases such as hypertension, diabetes, and breast cancer, have been associated with SD, which becomes more common with increasing age (Thomas et al., 2018 ▶). There are various treatments for SD, including medications (danazol, levodopa, amphetamines, and bupropion), behavioral (Exercise before sex, determining good times for sex, and psychotherapy), Complementary and alternative medicine (CAM) (acupuncture and herbal products), or a combination of these modalities (Lorenz et al., 2016 ▶; Burkman, 2012 ▶). The tendency to use complementary medicine in the management of disease is steadily increasing, and from over 15 years ago, the number of alternative medicine care provider visits has exceeded the number of primary care provider visits and women are the main users of these treatments (Burkman, 2012 ▶).* Ginkgo biloba* is one of the most popular herbal supplements used in the world (Nguyen and Alzahrani, 2019 ▶). Ginkgo trees are very large and on average 20 to 35 meters high, while some of these trees reach over 50 m in China (Royer et al., 2003 ▶). These trees are one of the oldest trees in the world and they are referred to as living fossils dating back over 250 million years (Dugoua et al., 2006 ▶). Extract from the leaves of *G. biloba* tree has compounds such as proanthocyanidins, phenolic acids, flavonoid glycosides, for example kaempferol, myricetin, quercetin and isorhamnetin, and the terpene trilactones, bilobalides and ginkgolides (van Beek and Montoro, 2009 ▶; van Beek, 2002 ▶). The leaves extremely have unparalleled ginkgo biflavones, polyprenols and alkylphenols (van Beek and Montoro, 2009 ▶). as a phytoestrogen modifies nitric oxide, soft tissue systems, and facilitates blood flow that are important for women's sexual reaction (Amiri et al., 2014). Up to now, some studies have evaluated the effect of GBE on SD (Malakouti et al., 2017 ▶; Amiri et al., 2014). During recent years, evidence-based practice as a way to support higher standards of care was emphasized by health care policy makers. Therefore, this study was performed to evaluate the current evidence for the efficacy of GBE on SD and its safety.

## Materials and Methods

**Data sources and search strategy**

In this review study, all international scientific and trustworthy databases such as PubMed, Scopus, Cochrane, Google Scholar, Web of science and Persian databases such as SID and Magiran were searched up to March 2020, to identify all the studies reporting the effect of *G. biloba* for effectiveness on sexual function and its safety. The search was performed using the keywords of Ginkgo, *Ginkgo biloba*, Complementary and alternative medicine, women sexual dysfunction, and male sexual dysfunction.

After finding all the articles with the keyword mentioned above, we manually reviewed each of the articles and eventually duplicated articles, articles with inappropriate content, and animal studies were removed.

The chosen studies met the following criteria to be contained in this review:

1. Study participants must be male or female. They had to be undertreated with ginkgo and the control groups received placebo or other medicines.

2.Clinical trials and randomized controlled trials (RCTs) that published in both English and 3. Persian languages; there was no limit to the year of publication.

3.Interventions consisted of ginkgo (*Ginkgo biloba*) 

4. Outcome measures were sexual function and sexual desire

**Figure 1:**
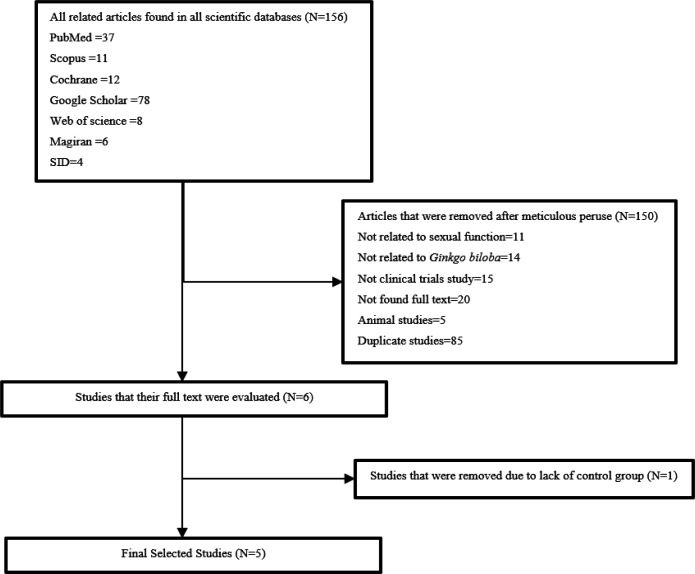
PRISMA flow diagram of article selection progress

Figure 1 shows how trials were selected.

**Study selection and data extraction**

After a general search, seemingly appropriate articles were collected by a researcher. All title and abstracts of collected articles were reviewed and after removing inappropriate articles, the remnant went to the next step and the full text of the selected articles was carefully evaluated. All of the above actions were reviewed again by a senior supervisor 

to prevent possible mistakes. Subsequently, all clinical trials and RCTs that tested the efficacy of *G. biloba* alone or in combination with other drugs to treat or improve sexual status were selected and arranged according to a predetermined checklist. The checklist included items such 

as Country, year, first author, study design, participant, intervention, comparison, tool, dropout, blinding method, outcome and adverse events. The disagreement between the researchers was resolved through consultation and discussion with a third researcher. The details of the selected articles are listed in Table 1.

**Table 1:** Summary of randomized clinical trials that tested *Ginkgo biloba* effects on sexual function

**Author year Country**	**Duration**	**Age (/Y)**	**Intervention mg**	**Type of control**	**Participants** **intervention**	**Participants** **control**	**Baseline** **comparability**	**Dropouts** ** (%)**	**Tools**	**Blinding method**	**Outcome(s)**	**Adverse events**
Malakouti et al. 2017 Iran	6 weeks	45 To 55	*Ginkgo biloba * 40 mg	placebo	N=60 postmenopausal women	N=60	N=116	3%	female sexual function index (FSFI)	double blind	The average of total sexual function scores in *Ginkgo biloba* and placebo groups enhanced from 17.5 (6.8) and 15.8 (5.7) to 21.6 (4.5) and 17.2 (4.2) respectively after intervention. Significant improvement in total (p<0.001) and all sub-domains scores of sexual function except pain (p<0.003) has been demonstrated in the covariance (ANCOVA) test analysis after intervention in Ginkgo biloba compared to placebo.	Nausea, Abdomen pain, Headache, Gripe
Amiri et al. 2014 Iran	One month	50 To 60	*Ginkgo biloba * 120-240 mg	placebo	N=40 postmenopausal healthy female volunteers	N=40	N=63	21.2 %	the Sabbatsberg Sexual Rating Scale (SSRS)	triple-blind	Significant improvements in sexual desire were observed after consuming *Ginkgo biloba* compared to the placebo (p=0.02).	Without any side effects
Meston et al. 2008 USA	8 weeks	18 To 65	*Ginkgo biloba* 300 mg/daily in long-term And single dose of 300 mg in short term group	placebo	N=134 sexually dysfunctional women (Chronic sample N=35 Acute sample N=99)	N=16	N=127	5.2%	the Female Sexual Function Index (FSFI), the Sexual Satisfaction Scale–Women (SSS-W), Vaginal photoplethysmography, arousometer	double-blind	Long-term GBE intervention did not significantly difference in arousal responses with placebo. 1) None of the short-term and long-term GBE alone showed any significant effect after the intervention.2) Also observed a significant effect in the placebo group in women with sexual concerns.	Not reported any side effects for ginkgo biloba
Wheatley, 2004 UK	12 weeks	18 To 65	*Ginkgo biloba* 240mg daily	placebo	N=11 patients with sexual impairment due to antidepressant drugs	N=13	N=21	12.5%	Sexual problems questionnaire (A new scale)	Triple-blind	From week 0 to week 6, no significant difference were found in any of the intervention groups.The non-blind follow-on period of another 6 weeks was completed by 6 participants on placebo and 7 on * Ginkgo biloba* , but there were no significant differences between week 18 and either week 0 or week 12.	Had to omit- Gastric pain/nausea- ‘Muzzy head’- Anaesthesia/paraesthesia ﬁngers and palpitations
Kang et al. 2002 South Korea	Two months	45 To 57	*Ginkgo biloba* 120-240 mg	placebo	N=19 patients with sexual impairment due to antidepressant drugs	N=18	N=25	32%	questionnaire	double-blind	No significant changes were found in any of the items related to sexual function at premedication or in the 2td and 4th weeks after intervention. After 8 weeks of intervention, only in one item (orgasm) were significantly difference found and even in the case of this item, the placebo group showed significant difference.	Gastrointestinal disturbance, Sedation ,headache , Increased oral intake

**Quality assessment of the included studies**

Oxford Center for Evidence-Based Medicine checklist for RCTs was used for assessing the quality of the chosen studies (Howick, 2011 ▶)( Table2). This instrument is designed in two parts that determine two segments; Internal Validity: containing six general queries regarding the way of patients assignment, matching and similarity of groups, equality of allocated treatment, intention-to-treat analysis and Losses to follow-up, effect size and blindness which was answered with three options Yes, No and Unclear.

**List of criteria for assessing the quality of studies, included:**

A: Was the assignment of patients to treatments randomized?

B: Were the groups similar at the start of the trial?

C: Aside from the allocated treatment, were groups treated equally?

D: Were all patients who entered the trial accounted for? – And were they analyzed in the groups to which they were randomized? (1: Losses to follow-up and 2: intention-to-treat)

E: Were measures objective or were the patients and clinicians kept "blind" to which treatment was being received? 

F: What were the results (Howick, 2011 ▶)"? 

## Results

**Study description**

From a total of 156 articles, 151 studies were excluded after peruse (Figure 1). At the end, five trials were included in our study and their key information is summarized in Table 1. Among these five studies, two RCTs were conducted in Iran, one study in the United States, one in the United Kingdom, and one in South Korea. All of these studies were conducted between 2002 and 2017, and a total of 475 people participated in these trials, 41 male and 434 female. 

The average age of the participants was 18 to 65 years. All participants received the *G. biloba* orally. Dosages were 40 mg to 300 mg of *G. biloba* daily and the duration of drug treatment was different from a single dose to 12 weeks. All studies reported placebo as a control group. Two RCTs investigated the effect of *G. biloba* on sexual function of antidepressants users and only these trials examined the effect of *G. biloba* in both males and females (Wheatley, 2004 ▶; Kang et al., 2002 ▶). Matson et al. (2008) ▶ investigated the effects of *G. biloba* in two groups of participants; Of the 99 participants in this study, 36 participants had sexual dysfunction due to the use of antidepressant medicines, and 63 participants did not use these drugs. Only this study examined both short and long term effects of *G. biloba* on sexual dysfunction in women. Two RCTs investigated the effects of *G. biloba* on sexual function in healthy postmenopausal women (Malakouti et al., 2017 ▶; Amiri et al., 2014). One of them investigated the effects of *G. biloba* tablet and aromatherapy inhaler combination on sexual function in females during postmenopausal period (Malakouti et al., 2017 ▶). The tools that used in these trials included the female sexual function index (FSFI), the Sabbatsberg Sexual Rating Scale (SSRS), the Sexual Satisfaction Scale–Women (SSS-W), Vaginal photoplethysmography, arousometer, Sexual problems questionnaire (A new scale). Specially, two studies have used the FSFI method to measure sexual function and their sexual scores were reported quantitatively (Malakouti et al., 2017 ▶; Meston et al., 2008 ▶). 

**Methodological quality**

The methodological quality of these studies is shown in Table 2. Randomization, blinding and dropouts were reported in all studies. Dropout rates ranged from 3 to 32% (Malakouti et al., 2017 ▶; Kang et al., 2002 ▶). In all trials, the treatment was equal in appearance between the intervention and placebo group. Only in one study, there was a statistically significant difference in terms of demographic characteristics between the control and intervention groups (Malakouti et al., 2017 ▶).

**Table 2:** Methodological assessment of study quality by Oxford Center for Evidence-Based Medicine checklist

No	**Studies**	**Criteria for methodological assessment of study quality**						
		A	B	C	D		E	F
					1	2		
1	Jamileh Malakouti et al. (2017) ▶, Iran	+	-	+	+	+	+	+
2	Amiri Mina et al. (2014), Iran	+	+	+	+	+	+	+
3	Cindy M. Meston et al. (2008) ▶, USA	+	+	+	+	+	+	-
4	David Wheatley et al. (2004) ▶, UK	+	+	+	+	+	+	-
5	Byung-JoKang et al. (2002), South Korea	+	+	+	+	+	+	-

**Outcomes**

After a meticulous review, we found that *G. biloba* can have positive effects on the sexual function of postmenopausal women, while evidence shows that it has no effect on the sexual function of antidepressant medicines users. Of the three RCTs that investigated the effect of *G. biloba* on antidepressant medicines users, none reported a statistically significant difference after the intervention (Meston et al., 2008 ▶; Wheatley, 2004 ▶; Kang et al., 2002 ▶). Two RCTs that examined the effect of *G. biloba* on sexual function of postmenopausal women, agreed with the efficacy *G. biloba* on sexual dysfunction and its safety (Malakouti et al., 2017 ▶; Amiri et al., 2014). Hence, future comprehensive RCTs are needed to demonstrate the definitive effects of *G. biloba* on sexual function.

**Adverse effects**

Only one study did not mention side effects of *G. biloba* (Meston et al., 2008 ▶). Headaches and gastrointestinal disturbance were among the adverse events mentioned in three trials (Malakouti et al., 2017 ▶; Wheatley, 2004 ▶; Kang et al., 2002 ▶).

## Discussion

In the present study effort to do a comprehensive review on RCTs that assesse the effects of *G. biloba* on sexual function. The results show that G. biloba can have positive effects on the sexual function of postmenopausal women, while evidence shows that it has no effect on the sexual function of antidepressants users. The RCTs report conflicting results, that could be due to differences in study methods, personal characteristics, environmental factors governing the study and bias. Due to the qualitative reporting of results in most of the RCTs included in this article, we were unable to do an extensive systematic review and meta-analysis study and could not find a very clear result. Therefore, implementation of further studies considering the effect of *G. biloba* on a variety of sexual function parameters and providing accurate all sub-domains scores of sexual function before and after the intervention, can make it possible to perform a comprehensive systematic review and meta-analysis study.

According to the results of this review study, it seems that *G. biloba* alone has a limited positive effect on sexual function, but combining it with other related herbs can increase its effectiveness. Ito et al. (2001) ▶ investigated the effect of Arginine Max (A combination of *G. biloba*, damyana, ginseng, L-arginine, multivitamins and minerals) on women over 21 years old and reported its positive effects. Five years later, they did the same RCT on premenopausal, perimenopausal, and postmenopausal women and reported improvements in sexual desire, sexual satisfaction, frequency of sexual desire and frequency of intercourse (Ito et al., 2006 ▶). Cohen and Bartlik, (1998) ▶ explained the mechanism of *G. biloba*'s effect on sexual activity. In summary, *G. biloba* improves sexual activity by increasing blood flow to the genitalia through inhibiting platelet-activating factor, inducing direct effects on prostaglandins to improve erectile function, creating relaxation in smooth muscle cells and affecting the nitric oxide (NO) system (Cohen and Bartlik, 1998 ▶). NO plays an important role in perception of sexual impulses in the brain and causing erections in penile and clitoris (Culotta and Koshland Jr, 1992 ▶). Therefore, *G. biloba* can have a positive effect on sexual function by increasing NO (Finkel et al., 1996 ▶; Marcocci et al., 1994 ▶). Elevated serotonin levels in depressed people are a possible mechanism for decreased sexual function (Higgins et al., 2010 ▶). Serotonin prevents the production of NO, and increasing it directly reduces sensation in the anatomical structures of the reproductive system (Higgins et al., 2010 ▶). Many antidepressants inhibit the autonomic nervous system by acting on cholinergic and alpha-1-adrenergic receptors, thereby inhibiting normal sexual function (Higgins et al., 2010 ▶). Therefore, all the findings of these studies are consistent with our results in this review study on the effect of the *G.* *biloba* on the sexual function of postmenopausal women and its ineffectiveness in the antidepressants users. Finally, we suggest that due to the general popularity of the use of medicinal plants among people around the world, *G. biloba* can be used in combination with other plants that improve sexual function. Of course, future comprehensive RCTs are necessary to demonstrate the definite positive effects of *G. biloba* on sexual function.

We concluded that *G. biloba* has limited positive effects on sexual function. However, the average methodological quality of the primary studies, the total number of RCTs and the total sample size were too limited to find a clear result. Subsequent trials should be done to confirm effects of *G. biloba* on sexual function.
